# The Dual Role of Platelets in the Cardiovascular Risk of Chronic Inflammation

**DOI:** 10.3389/fimmu.2021.625181

**Published:** 2021-04-01

**Authors:** Carlos Zamora, Elisabet Cantó, Sílvia Vidal

**Affiliations:** Inflammatory Diseases, Institut de Recerca de l'Hospital de la Santa Creu i Sant Pau, Biomedical Research Institute Sant Pau (IIB Sant Pau), Barcelona, Spain

**Keywords:** platelet, chronic inflammation, cardiovascular risk, leukocytes, systemic inflammation

## Abstract

Patients with chronic inflammatory diseases often exhibit cardiovascular risk. This risk is associated with the systemic inflammation that persists in these patients, causing a sustained endothelial activation. Different mechanisms have been considered responsible for this systemic inflammation, among which activated platelets have been regarded as a major player. However, in recent years, the role of platelets has become controversial. Not only can this subcellular component release pro- and anti-inflammatory mediators, but it can also bind to different subsets of circulating lymphocytes, monocytes and neutrophils modulating their function in either direction. How platelets exert this dual role is not yet fully understood.

## Introduction

Systemic inflammation (SI) has been described as a consequence of increased levels of the circulating pro-inflammatory mediators that activate endothelial cells (EC). Endothelial activation is part of a normal immune system defense, but a prolonged inflammatory stimulus induces a sustained endothelial activation/dysfunction that is often associated with atherogenesis and cardiovascular events. The role of SI on cardiovascular (CV) risk has been explored in autoimmune diseases. Systemic lupus erythematosus (SLE)-like mouse models display endothelial dysfunction and cardiac hypertrophy, mediated through IL-6 and IL-1α (1). In these patients, it has been shown that B lymphocyte stimulator induced apoptosis of endothelial progenitors cells and EC (2). In addition, it has been suggested that autoantibodies play a role in endothelial dysfunction, possibly by modulating the adhesion of neutrophils (3, 4). In a model of arthritis, endothelial dysfunction was only observed in rats with a persisting imbalance between NOS and COX-2 pathways, higher plasma levels of IL-1β and tumor necrosis factor-α (TNF-α) (5). The presence of diabetes mellitus type 2 in patients with metabolic syndrome impairs the endothelial function (6). In a model of SI, the levels of endothelin-1 and endocan are related to endothelial dysfunction (7). However, there are some circumstances in which the relationship between SI and endothelial dysfunction is less clear. In Systemic Inflammatory Response syndrome, the levels of dysfunctional EC were associated with mortality and organ dysfunction independently of inflammatory markers (8).

The link between inflammation and endothelial dysfunction has been confirmed by the inhibition of molecules related to SI. Anti-TNFα antibodies reduces sE-selectin and sVCAM expression (9) and decreases endothelium-dependent relaxation (10, 11). EC treated with etanercept revert the apoptosis induced by TNF-α (12). JAK inhibitors improve endothelium dependent vasorelaxation, endothelial cell differentiation and lipoprotein profiles, while decreasing pro-inflammatory cytokines in SLE-like syndrome (13). Glucocorticoids decrease IL-1β and TNF-α levels, improving the function of endothelium in rheumatoid arthritis (RA) (14). Patients with CV risk factors had increased levels of IL-1β and its gene expression signature and blocking IL-1β with canakinumab was observed to prevent recurrent cardiovascular disease (CVD) (15). Agents with nucleoside triphosphate hydrolase activity decrease platelet-leukocyte-endothelium interaction, the transcription of pro-inflammatory cytokines, microvascular platelet-neutrophil aggregate sequestration, activation marker expression on platelets (PLTs) and neutrophils contained in these aggregates, leukocyte extravasation, and organ damage (16). Furthermore, dihydroartemisin inhibits the occludin downregulation induced by TNF-α, improving the permeability of EC (17) and the inhibition of cannabinoid receptors reduces leukocyte-adhesion and improves microvascular blood flow (18). The pre-treatment of primary cultured human umbilical vein endothelial cells (HUVECs) with sevoflurane reduces ICAM-1 (intercellular adhesion molecule-1) and VCAM-1 (vascular cell adhesion molecule-1) IκBα, and NF-κB activation, and blocks the adhesion of leukocytes (19).

Products with anti-inflammatory properties can improve endothelial function. Lactobacillus plantarum 299v supplements decreased inflammatory markers (20), while the active form of vitamin D diminishes IL-6 secretion and increases the angiogenic capacity of myeloid angiogenic cells via CXCL10 down-regulation (21).

## Features of Endothelial Dysfunction

The inflammatory phase that leads to endothelial dysfunction is initiated by TNF-α and subsequently amplified by IL-1, IL-6 and downstream mediators. Endothelial dysfunction refers to the failure by ECs to perform their physiological functions, often due to a maladaptive response to pathological stimuli. The phenotypic features of endothelial dysfunction include the upregulated expression of endothelial leukocyte adhesion molecules (ELAMs) [E-selectin, ICAM-1 and VCAM-1]. On leukocytes, activated ECs induce the affinity of counter-receptors for ELAMs and secrete and display chemokines on the luminal surface. Endothelial dysfunction also includes a compromised barrier function, the secretion of microvesicles, an increased vascular smooth muscle tone, and the increased production of vasoconstrictor substances, the reduced resistance to thrombosis via platelet aggregation and oxidative stress upregulation (22–24). It was described that high levels of IL-8 and TNF-α up-regulate CX3CR1 expression on platelet-monocyte aggregates, increasing adhesion to activated endothelium (25). In mouse models, increased IL-17 was associated with reactive oxygen species formation, circulating inflammatory leukocytes and endothelial dysfunction (26), while higher levels of resistin, TNF-α, IL-1β, and MMP-9 expression were associated with the levels of inflammatory infiltrates in artery walls (27).

Beyond the activation of the well-known signaling cascades, the stimulation of EC induces gene expression via microRNAs (miRNA) and epigenetic modifications. The overexpression of miR100 in ECs attenuates leukocyte-endothelial interaction, represses the mammalian target of rapamycin complex 1 signaling, stimulating endothelial autophagy, and attenuates NF-κB signaling. Local miR100 expression is inversely correlated with an inflammatory cell content (28). miR181b inhibits downstream and upstream NF-κB signaling in response to activation (29), while the NF-κB target genes (VCAM-1, ICAM-1, E-selectin, and tissue factor) (30) and miR223 are associated with HUVEC dysfunction (31). Additionally, IFN-α, through miR155, promotes an endothelial dysfunction signature in HUVECs characterized by transcription suppression and the mRNA instability of eNOS and by the upregulation of MCP-1 and VCAM-1 and enhanced neutrophil adhesion (32).

Endothelial microparticles (MPs), shed as a result of the activation of EC are considered a source of important information on the status of ECs and vascular function (33, 34). Circulating levels of endothelial MPs reflect a balance between cell stimulation, proliferation, apoptosis, and cell death (35) and are increased by inflammatory stimuli, mediated by the activation of NF-κB and associated with oxidative stress intensity (36, 37). Endothelial MPs are increased in autoimmune diseases (38) and serve as markers for vascular dysfunction and their effects depend on their cargo and on the surface molecules. Recently, levels of endothelial MPs have been associated with disease activity in SLE patients and CV risk (39).

MPs from RA patients had higher expression of TNF-α on the surface compared to healthy donors (HD), increasing apoptosis and autophagy levels on EC and correlating with clinical RA activity (40).

## Platelets and Systemic Inflammation

PLTs have come to be recognized as active players in SI. After activation, PLTs participate in the vasculature inflammation and damage, atherogenesis and thrombosis (41–45). Wide ranges of stimulus are able to activate PLTs. The strong PLT activation was achieved with the ligation of the agonist thrombin, collagen and ADP to the PLTs receptors: protease-activated receptor 1, GPVI and P2Y1 or P2Y12 respectively (46–48). Other non-classical pathways are able to activate PLTs due to the expression of Toll-like receptors (TLR), TNF-α receptor, IL-1β receptors and C-type lectin-like receptors (49–53). Some autoantibodies presents in autoimmune disease patients such as anti-citrullinated protein, anti-β2 glycoprotein I and anti-D4GDI have also the ability to induce PLT activation through FCγRIIa (54–56).

However, PLTs also participate in the resolution of inflammation as anti-inflammatory elements. How PLTs sense the signal to exert pro- or anti-inflammatory functions is not yet fully known. However, it is known that PLTs exert their functions by releasing soluble factors and interacting with cells. The dual role of PLTs in inflammation (57, 58) may be the result of differences in the PLT packing of molecules, activated-dependent release by different stimuli, the kinetics of release and the *de novo* synthesis of soluble factors and their binding to certain molecules on the surface of leukocytes.

### Pro-inflammatory and Anti-inflammatory Soluble Factors Released by PLTs

Some of the released factors of PLTs are synthesized *de novo*, whereas others are stored and are secreted from granules as pro-thrombotic, immunoregulatory molecules and growth factors immediately after activation. Molecules from dense granule components contribute to hemostasis and coagulation. Molecules from α-granules contain multiple cytokines, mitogens, pro- and anti-inflammatory factors and other bioactive molecules that are essential regulators in the complex microenvironment (Table 1).

**Table 1:** Evidences of dual role of expressed/secreted platelet factors.

**Factor**	**Pro-inflammatory**	**Anti-inflammatory**
PF4	• Monocytes: ↑ phagocytosis (59) ↑ respiratory burst (59) ↑ pro-inflammatory cytokines (59) • Neutrophils: ↑ NETs formation (60) • Endothelial cells: ↑ Leukocyte recruitment (60)	• T lymphocytes: ↓proliferation (61) ↓Th17 differentiation (62) ↓granzyme B (61)
IL-1	↑ endothelial activation (63, 64) ↑ EC damage (63) ↑ neutrophil adhesion (63)	
P-selectin	• Monocytes: ↑ pro-inflammatory cytokines (65) • Neutrophils: ↑ NETs formation (66)	•*In vitro*: ↓neutrophil adhesion to activated EC (67)
sCD40L	Plasmatic levels correlated CV risk and pro-inflammatory cytokines (68, 69) ↑ B cell isotype switching (70)	• Monocytes: ↓TNF-α (71) ↑IL-10 (71) Correlation with IDO, Treg (68)
TGF-β		• T lymphocytes: ↓T cell function and proliferation (72, 73) ↓Th1, Th17 response (72) ↓IFN-γ production (72, 73) ↓granzyme B and perforin (73) ↑Treg differentiation (72)
HMGB1	• Monocytes: ↑ migration and accumulation in tissues (74, 75) ↓apoptosis (74) • Neutrophils: ↑ NETs formation (76, 77)	

Platelet Factor 4 (PF4, also called CXCL4) is the most abundant protein secreted by activated PLTs and is deposited on endothelium. Higher levels of circulating PF4 have been observed in patients with chronic inflammation (78–80). PF4 increases phagocytosis, respiratory burst, survival and the secretion of inflammatory cytokines in monocytes (59). PF4 blocking reduces the inflammation of vasculature and CV events by reducing leukocyte recruitment and the generation of neutrophil extracellular traps (NETs) by neutrophils (60, 81, 82).

PF4 also acts as an anti-inflammatory factor on T lymphocytes (83), limiting Th17 differentiation by suppressing RORγ expression (62). A lack of PF4 induces the rejection of cardiac transplantation by increasing levels of IL-17 and T cell mediated inflammation. We has observed that PF4 decreases T lymphocyte proliferation and granzyme B expression in CD8+ T lymphocytes (61), explaining how higher levels of PF4 in a malignant context can limit T lymphocyte stimulation (61).

Stimulated PLTs are able to secrete and store IL-1β (63). PLT levels were closely associated with plasmatic IL-1β levels (15). This cytokine activates HUVECs, inducing neutrophil adhesion and endothelium damage (63). The co-culture of PLTs from SLE with HUVECs increased EC damage and inflammatory marker expression in an IL-1β dependent manner (64). In experimental models of inflammation, IL-1α secreted by activated PLTs also played a crucial role in SI (84–86).

The soluble P-selectin secreted from α-granules is also implicated in inflammatory responses. P-selectin from activated PLTs induces the release of 3-10 folds of inflammatory cytokines by monocytes (65) and also promotes NETs formation (66). Elevated levels of circulating soluble P-selectin may contribute to early vascular disease by promoting the adhesion of leukocytes to the endothelium (87). However, soluble P-selectin can prevent *in vitro* adhesion of neutrophils to activated endothelium (67).

Circulating soluble CD40L (sCD40L) is secreted mainly by activated PLTs. In human immunodeficiency virus (HIV) patients, sCD40L levels correlated with pro-inflammatory cytokines (68). Moreover, PLTs support B cell isotype switching through CD40L-CD40 binding (70). In patients with an increased CV risk plasmatic sCD40L was increased and correlated with disease activity and with pro-inflammatory cytokines (69). The addition of thrombin-activated PLTs to TLR-stimulated monocytes has been seen to reduce TNF-α and IL-6 secretion and induce IL-10 production, and were abolished by blocking sCD40L (71). In HIV, sCD40L levels were correlated with IDO enzymatic activity and Treg frequency, in addition to induced Treg expansion and differentiation (68).

TGF-β is a potent anti-inflammatory factor, produced mainly by PLTs, which suppresses T lymphocytes function and is involved in Treg differentiation. The culture of CD4+ T lymphocytes with PLTs enhances Th1 and Th17 cytokine production but the TGF-β secreted by PLTs activates Treg suppressing Th1 and Th17 response (72). PLTs inhibit CD4+ and CD8+IFN-γ production, proliferation and granzyme B and perforin expression in a TGF-β dependent manner (73).

PLTs also release the damage-associated molecular pattern molecule high-mobility group box 1 (HMGB1) contributing to thrombosis process (87) promoting monocytes migration, suppressing monocyte apoptosis via TLR4-ligation and the monocyte accumulation at the site of vascular thrombosis (74, 75) and promote NETs formation (76, 77).

### Binding of PLTs to Leukocytes and Endothelial Cells

The interaction of PLTs with leukocytes and EC contributes to SI by favoring the arrest of leukocytes on endothelium, the production of inflammatory cytokines and NETs formation. Under certain circumstances, the binding of PLTs to leukocytes decreases the inflammatory response, participating in the resolution of thrombo-inflammation (Table 2).

**Table 2:** Effects of PLT binding to other cells.

	**Ligands of interaction**	**Effect on bound cells**
Endothelial cells	GPIIB/IIIa- ICAM1 CD40L-CD40	Adhesion (88) Expression E-selectin VCAM-1 (89) Secretion IL-8 MCP-1 (89)
Neutrophils	GPIβα-Mac-1 P-selectin-PSGL-1	Arrest to endothelium (90–92) NETs formation (66, 93)
Monocytes	P-selectin-PSGL-1 GPIβ-CD11b CD147-CD147 CD40L-CD40	Secretion MCP-1, IL-8, TNF-α, MMP9 (94, 95) Expression TF (96) Differentiation M1 macrophages (97) Secretion IL-10 (71, 98, 99) Reduction TNF-α, IL-1β (71, 99)
Lymphocytes	P-selectin-PSGL-1 P-selectin-ALCAM	•*(SLE) B cells*: Upregulation CD86, BAFF (100) Secretion IL-10 (100) • *(Psoriasis) CD4+:* Secretion IL-17 (101) • *Healthy donors:* Less proliferation (102–105) Less pro-inflammatory cytokines (102–105)

Under inflammatory stress, PLTs have a firm adhesion to endothelium via GPIIB/IIIa-ICAM-1 in a fibrinogen dependent manner (88). CD40L expressed by PLTs induces the expression of E-selectin, VCAM-1 and ICAM-1 on endothelium and the secretion of IL-8 and MCP-1 (89), increasing the recruitment of leukocytes.

Although the P-selectin-PSGL-1 (P-selectin glycoprotein 1) axis is essential to the binding of PLTs to leukocytes, other pathways are involved in this process: P-selectin-ALCAM, GPIβα-Mac1, CD40L-CD40, P-selectin-CD15, JAM-C-Mac1, TREM1L-TREM1, CD36-trombospondin-CD36, and CD147 pathway (94, 106, 107).

The interaction of PLTs with neutrophils through GPIβα-Mac-1 and P-selectin-PSGL-1 is crucial for the development of thrombo-inflammation and vascular damage by arresting neutrophils to endothelium and the induction of NETs formation (66, 90–93). The neutrophil-platelet aggregates can also be seen in tissues in acute coronary syndrome and the skin of psoriatic patients (108, 109). Stimulated TLR4 PLTs, through the binding with neutrophils, induced robust neutrophil activation and formation of NETs (93). Induced NETs formation by PLTs was abolished blocking their binding with neutrophils (110). The increase of neutrophil-platelet aggregates in the circulation of autoimmune disease patients correlates with neutrophil activation (111) and vascular abnormalities (112) which was abolished with intravenous immunoglobulins plus prednisolone treatment (112). However, even all the current evidences supports that interaction of PLTs with neutrophils are involved in inflammatory process and vasculature damage, this interaction could have also anti-inflammatory consequences depending on the neutrophil status. The addition of PLTs to previously stimulated TLR-neutrophils downregulates degranulation markers expression and the secretion of elastase (113).

Although monocyte-PLT aggregates are increased in CVD (114), the interaction of PLTs with monocytes have pro- or anti-inflammatory consequences depending on the experimental assay and the activation status of monocytes. As pro-inflammatory consequence, thrombin-activated PLTs through P-selectin-PSGL-1 binding induces the expression of MCP-1 and IL-8 in resting monocytes (95). P-selectin expressed on the surface of PLTs induced a rapid tissue factor expression by monocytes (96). The binding of PLTs to monocytes through GPIβ-CD11b induces a M1 macrophage phenotype that produce TNF-α (97). Via CD147 axis, RA patients had more circulating intermediate monocytes-PLTs aggregates, increasing the TNF-α and MMP-9 secretion (94). Autoimmune patients with a higher CV risk have more monocyte-PLT aggregates and its associated with the activated state of monocytes (115). As anti-inflammatory consequences, it has been observed that the binding of PLTs to activated monocytes induces IL-10 production and decreases TNF-α and IL-1β production, and were abolished by the blocking of P-selectin-PSGL-1, CD40L-CD40 axis and Ca_2_+ chelator (71, 98, 99).

Although the binding of PLTs to lymphocytes has preferably anti-inflammatory consequences, it was demonstrated a contribution in inflammatory process. High levels of lymphocytes-PLTs aggregates were observed in SLE and psoriasis. In SLE, lymphocytes-PLTs aggregates had an up-regulation of CD86, B cell activation factor receptor and IL-10 production and correlated positively with plasmatic levels of IgG, IgA, IL-10, sCD40L and renal manifestation, and correlated negatively with IgM levels (100). In psoriasis, the IL-17+ CD4+ had higher levels of bound PLTs and anti-TNF-α drugs normalize the numbers (101), while in HIV there are more lymphocytes-PLTs aggregates and are associated to D-dimer levels, increasing the CV risk (116).

We observed that CD4+ T lymphocytes-PLT aggregates had a reduced proliferation and production of pro-inflammatory cytokines. A less severe phenotype and a decreased CV risk was observed in RA patients with higher levels of circulating CD4+T lymphocytes-PLTs aggregates (102). The addition of PLTs to lymphocytes from RA synovial fluids decreased their proliferation and the secretion of IFN-γ, IL-17, and increased IL-10 production (103). PLTs MPs cultured with Tregs prevented the differentiation into IL-17– and IFN-γ-producing cells in a P-selectin dependent manner (104). The co-cultures of CD4+ CD25- T cells with PLTs induced Tregs and this effect was abolished by the blocking of glycoprotein A repetitions predominant (105). In HIV, the aberrant function of CD8+ T lymphocytes was abolished when these cells were co-cultured with PLTs from HD, implying that direct contact with PLTs and TGF-β secretion contributed to this functional improvement (117). In later stages of experimental autoimmune encephalomyelitis (EAE), there was an increase CD4+ T lymphocytes-PLTs aggregates through the interaction of P-selectin-ALCAM, down-regulating their activation, proliferation and the production of IFN-γ, crucial for the spontaneous resolution of EAE. The blocking of CD4+-PLT aggregates exacerbate EAE (118).

### Platelet to Lymphocyte Ratio in Systemic Inflammation

The platelet to lymphocyte ratio (PLR) has emerged as a reliable marker of SI. Although elevated counts of PLTs and low counts of lymphocytes *per se* has been associated with worse prognosis of CVD, increase CV mortality and morbidity and SI (119, 120), PLR predicts better the outcomes of CVD. The role of PLR as an independent marker in CVD and SI has been extensively reviewed (121, 122). In patients with chronic inflammatory diseases, PLR is elevated and it correlates with markers of SI (122–125).

However, there are confounding factors that alter PLR. Sex and ethnic origin also modulates PLR (126) as well as drugs that affect blood cell maturation in the bone marrow (127, 128). Other confounding factors of PLR may be the technical limitations of PLR measurements, such as EDTA dependent agglutination (129, 130).

## Conclusions

PLTs have been considered to play a pro-inflammatory role in SI. However, their binding to leukocytes and EC and the secretion of immunomodulatory molecules during activation also have anti-inflammatory consequences. Different effects were observed with platelets from healthy donors or chronic inflammatory patients. In addition, the binding to each subpopulation of leukocytes has distinctive consequences. Further research is necessary to reveal how platelets exert their dual function.

## Author Contributions

CZ and EC: literature review, manuscript preparation. SV: oversight, editing, and planning. All authors contributed to the article and approved the submitted version.

## Conflict of Interest

The authors declare that the research was conducted in the absence of any commercial or financial relationships that could be construed as a potential conflict of interest.
